# Feasibility and Reliability of a Questionnaire to Assess the Mode, Frequency, Distance and Time of Commuting to and from School: The PACO Study

**DOI:** 10.3390/ijerph17145039

**Published:** 2020-07-13

**Authors:** José Manuel Segura-Díaz, Álvaro Rojas-Jiménez, Yaira Barranco-Ruiz, Berta Murillo-Pardo, Romina Gisele Saucedo-Araujo, María Jesús Aranda-Balboa, Manuel Herrador-Colmenero, Emilio Villa-González, Palma Chillón

**Affiliations:** 1 PROFITH “PROmoting FITness and Health through Physical Activity” Research Group, Department of Physical Education and Sports, Faculty of Sport Sciences, Sport and Health University Research Institute (iMUDS), University of Granada, 18071 Granada, Spain; imponator@correo.ugr.es (Á.R.-J.); rgs@ugr.es (R.G.S.-A.); mjab@ugr.es (M.J.A.-B.); mhc@ugr.es (M.H.-C.); pchillon@ugr.es (P.C.); 2 PROFITH “PROmoting FITness and Health through Physical Activity” Research Group, Department of Physical Education and Sports, Faculty of Education and Sport Sciences, Sport and Health University Research Institute (iMUDS), University of Granada, 52071 Melilla, Spain; ybarranco@ugr.es (Y.B.-R.); evilla@ugr.es (E.V.-G.); 3 Department of Music, Plastic and Bodily Expression, University of Zaragoza, 50009 Zaragoza, Spain; murillop@unizar.com; 4 La Inmaculada Teacher Training Centre, University of Granada, 18071 Gradana, Spain

**Keywords:** psychometric properties, self-reported, active transport, youths

## Abstract

Active commuting to and from school has several health implications. Self-reporting is the most common assessment tool, but there is a high heterogeneity of questionnaires in the scientific literature. The purpose of this study was to analyse the feasibility and reliability of the Spanish “New Version of Mode and Frequency of Commuting To and From School” questionnaire in children and adolescents. A total of 635 children (5–12 years old) and 362 adolescents (12–18 years old) filled out the questionnaire twice (14 days apart). Feasibility was evaluated using an observational checklist. The test-retest reliability of the “New Version of Mode and Frequency of Commuting To and From School” questionnaire and the distance and time to school were examined using the kappa and weight kappa coefficient (κ). No misunderstanding of questions was reported. The time to complete the questionnaire was 15 ± 3.62 and 9 ± 2.26 min for children and adolescents, respectively. The questionnaire showed substantial and almost perfect kappa coefficients for the overall six items (*k* = 0.61–0.94) in children and adolescents. The “New Version of Mode and Frequency of Commuting To and From School” questionnaire is a feasible and reliable questionnaire in Spanish children and adolescents.

## 1. Introduction

Obesity in childhood and adolescence is a pressing public health problem worldwide [1]. It is associated with a higher risk of some adulthood cardiometabolic diseases and mortality [2,3]. In Spain, the prevalence of obesity in young people increased between 1983 and 2011 from 13.9% to 22.2% in boys aged 10 to 14 years old [4], being higher than in other European countries. The percentages of overweight/obesity in European children aged 7 to 14 years old, were above 18.9% in Germany, 19.5% in Ireland, 12.3% in Poland, 15.0% in Sweden, 17.4% in Switzerland and 32.1% in Spain [5]. This continuing intensification of the obesity rates is producing a higher exploitation of the health services such as hospital inpatient stays and the use of general practitioners, resulting in economic consequences in many countries [6,7,8]. Increasing physical activity (PA) in young people could be a possible low-cost strategy to curb rising obesity rates [9]. Currently, according to the data from the Spanish National Health Survey, the proportion of physically active children (i.e., achieving 5 or more days of 60 min of Moderate-to-Vigorous Physical Activity -MVPA-) is 31.0% for males and 15.0% for females from 3 to 18 years old [10]. Additionally, worldwide between 30.0%–40.0% of children and adolescents meet these recommendations [11,12].

Active commuting to and from school (ACS), i.e., traveling to and from school by walking or cycling, is a promising strategy to increase the daily PA in youths [13]. In a review conducted by Larouche et al., the relation between ACS and daily PA was assessed showing 81.6% of the studies with significantly higher PA levels in active children and adolescents compared to not actives [14]. This active behaviour produces improvements in several health parameters, including greater speed-agility, body composition and cardiorespiratory outcomes [15,16], with the best results seen from using bicycles [17]. In a recent systematic review, ACS was positively associated with perceptions of safety, walkability, and neighbourhood social interaction in children [18]. In addition, walking and cycling entail a low-cost, immediate, and normative activity that could reduce costs in the National Health Service, reducing air and noise pollution, traffic congestions and CO_2_ emission in the cities [19]. Finally, regarding the psychological benefits of the ACS, a study suggested that children who commuted more times weekly to school seemed to have a lower level of stress than the less active children [20].

Most of the scientific studies focused on ACS have used a questionnaire to evaluate this behaviour; however, according to the last systematic review (published in 2014) that evaluated the self-reported modes and frequency of commuting to school in young people, only a 33% of the studies indicated the feasibility, validity and reliability of the questionnaires used [21]. Besides, several studies showed different questions and methods to be compared [22,23,24]. Due to the few studies evaluating feasibility, our study provides more information about an important gap that exists in the scientific literature with reference to the evaluation of the feasibility in questionnaires. For example, in a study from the United States, the questionnaire included questions about children’s school travel behaviour [22]; in a study from Canada, questions about the mode of transportations to school were included [23] and in Germany, a single question about the usual mode of ACS was considered [24]. The “Mode and Frequency of Commuting To and From School” questionnaire was suggested by Herrador-Colmenero et al. based on a deep analysis of questionnaires in the scientific literature [21] and then, it was validated in children and adolescents using accelerometry as the gold standard by Chillón et al. [25]. However, the questionnaire included in the study of Chillón et al., did not evaluated the validity of any questions based on distance or time to commute to or from school, although these variables have been shown to be important factors related to this behavior [26,27]. Thus, this study aimed to formally evaluate the feasibility (i.e., time of completion and understanding) and the reliability (i.e., the consistency, coherence and constancy) of the “New Version of Mode and Frequency of Commuting To and From School” questionnaire in a sample of Spanish children and adolescents.

## 2. Methods

### 2.1. Design and Participants

This is a test-retest study, where the participants completed the “New Version of Mode and Frequency of Commuting To and From School” questionnaire twice, 14 days apart: between February–May 2016 (*n* = 712) and between March–April 2018 (*n* = 285). The descriptive characteristics of the participants are shown in Table 1.

**Figure 1:**
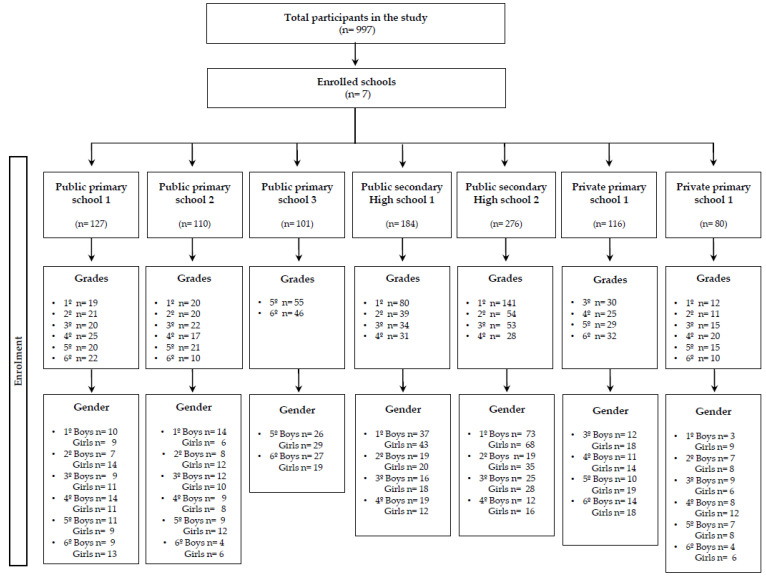
Flow diagram of the study participants.

The test-retest is an appropriate study design to evaluate the reliability and asking students about potential problems to understand the questionnaires and the time used for it are practical issues to evaluate the feasibility. A total sample of 997 participants, including 635 children (5–12-years-old, i.e., grade 1st to 6th) and 362 adolescents (12–18-years-old, i.e., grade 7th to 12th) from Granada (South of Spain) were invited to participate in this study. The flow diagram of the study participants is shown in Figure 1. The sample of participants in this study was homogeneous regarding gender, both for children (*n* = 626; 52% girls) and adolescents (*n* = 360; 51% girls). The mean age of the children was 9.77 ± 1.86 years old (*n* = 635), whereas adolescents presented a mean age of 14.80 ± 1.48 years old (*n* = 362). Granada is the capital city of the province of Granada, in the autonomous region of Andalusia (Spain). Out of the initial 635 children and 362 adolescents who were invited to take part in the study, 414 children and 298 adolescents were excluded from the feasibility analysis since their starting and/or ending time were missing. Participants who answered more than two response options or left the response option blank both in the test or retest were excluded from the reliability analysis (Question 1 º *n* = 204, Question 2 º *n* = 201, Question 3 º *n* = 0, Question 4 º *n* = 0, Question 5 º *n* = 172 and Question 6 º *n* = 153).

### 2.2. Procedure

The study was explained to the participants before starting, and parents or tutors signed an informed consent. We conducted a cross-sectional study as part of the previous phase test of the PACO study (Pedalea y Anda al COlegio/Cycle and Walk to School), whose main purpose was to design and analyse questionnaires (e.g., the “New Version of Mode and Frequency of Commuting To and From School” questionnaire) to be implemented later in an intervention study to promote the mode of commuting to school and PA levels [28]. For the sample selection, firstly, the main researcher of the research group selected primary and secondary schools through a purposeful sampling method, in accordance with its previous collaboration in other studies. Secondly, the research staff contacted with both the principal and physical education teacher of each school to obtain informed consent. Participants were selected from three public primary schools (School 1 (children = 152), School 2 (children = 135) and School 3 (children = 101), two secondary public schools (High School 1 (adolescents = 184), and High School 2 (adolescents = 178), and two private primary schools (School 1 (children = 126) and School 2 (children = 121). The schools had a medium socio-economic level—data collection year was obtained from the Tax Agency Spanish Public—(https://www.agenciatributaria.es/) and were located in an urban environment. Two previous meetings were conducted with the Physical Education teacher and headmaster of the schools to inform about the research project and they accepted to participate. Children delivered informed consents to their parents to be signed. Children and adolescents who completed the “New Version of Mode and Frequency of Commuting To and From School” questionnaire, in both test and retest, were included in the final analysis (children, *n* = 504; adolescents, *n* = 321).

All measurements were taken under similar conditions regarding the weekday (Tuesday, Tuesday or Thursday from 8:15 am to 3:15 pm) and the evaluators (4 evaluators); the seven schools belonged to the same region (Granada) and had similar weather conditions (variable and temperate climate). Regarding weather data, the mean temperature registered in Granada was 22° during 2016–2018 according to the Spanish Meteorological State Agency (www.aemet.es). Granada has an average elevation of 738 m (2421 ft.) above the sea level and is the 13th largest urban area of Spain.

This study followed the ethical standards recognised by the Declaration of Helsinki, and the study protocols were approved by the Ethics Committee of the University of Granada (Granada, Spain; case number 162/CEIH/2016) and the Regional Ministry of Education of Andalucia.

### 2.3. Instruments

#### 2.3.1. Feasibility Sheet

**Figure 2:**
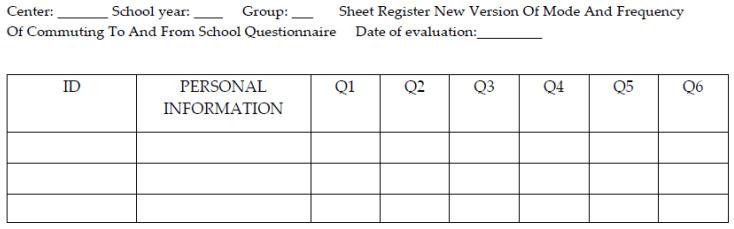
Sheet Register of the New Version of Mode and Frequency of commuting to and from school questionnaire for Students’ doubts. Notes: ID, Identification number; Q, Question.

Feasibility was examined with an observational sheet, where doubts that arose during the questionnaire fulfillment were recorded, as well as the time spent to complete the questionnaire (Figure 2).

During the process, 2 researchers supported participants to complete the questionnaire and the researcher wrote every doubt from the participants to collect them. To know the identity of the person who had doubts, their ID and their personal information was written. The Spanish observation sheet has been included in supplemental Figure S1.

#### 2.3.2. The New Version of Mode and Frequency of Commuting To and From School Questionnaire

The “Mode and Frequency of Commuting To and From School” questionnaire, is a 4-item self-report instrument previously validated by Chillón et al. [25] designed to evaluate the mode and weekly frequency of ACS in children and adolescents. The four questions included in the questionnaire were: (1) How do you usually get to school?; (2) How do you usually get home from school?; (3) How did you get to school each day?; (4) How did you get home from school each day?; all the questions were provided with these answers: walk, cycle, car, motorcycle, school bus, public bus or metro/train/tram, or others (the mode description was required).

In addition to the previous questionnaire that was validated, two relevant additional questions about distance and time not previously validated were evaluated in the “New Version of Mode and Frequency of Commuting To and From School” questionnaire: (5) How far do you live from school?; provided these answers: < 0.5 km, 0.5 km to <1 km, 1 km to <2 km, 2 km to <3 km, 3 km to <5 km and 5 km or more; (6) How long does it take to get to the school since you leave your house?; provided these answers: <15 min, 15 min to <30 min, 30 min to <60 min and 60 min or more. 

The Spanish and English versions of the questionnaires are freely available: http://profith.ugr.es/pages/investigacion/recursos/cuestionarioespan_ol.

### 2.4. Statistical Analysis

A descriptive statistic was performed with percentages for categorical variables and means and standard deviations for continuous variables. The feasibility was assessed considering the time needed to complete the questionnaire and recording the questions that were not understood by the participants. The differences in the time spent to complete the questionnaire only in the test were analysed using a dependent *t* student test with a Bonferroni adjustment for multiple comparisons. Both paired samples were used to check the time spent in the test by the same children and adolescents and unpaired samples when comparing children and adolescents, different age groups and gender. Age-related differences on time have been included in supplemental Table S2. The test-retest reliability was calculated using the kappa coefficient for the “usual mode to and from school” and for the “weekly mode to and from school” items, and the weighted kappa coefficient (κ) for the “distance and time to school” items. The weighted kappa coefficient is appropriate to calculate the degree of agreement of categorical variables in which there is a graduation order or ordinal variables [29]. The analysis was performed separately for children and adolescents, for different age groups (6–7, 8–9, 10–11, 12–13, 14–15 and 16–17-years-old) and boys and girls. To classify the results obtained from the weighted kappa, cut points proposed by Colton et al. [29] were used: < 0.0 = poor; 0.00–0.20 = light; 0.21–0.40 = correct; 0.41–0.60 = moderate; 0.61–0.80 = substantial; 0.81–1.00 = almost perfect. The Fujitsu fi-7160 scanner and the Data-scan software version 5.7.7 were used to read the questionnaires and create a database. All analyses were performed using the SPSS v.22.0 program (SPSS Inc, Chicago, USA) for Windows. The level of significance was set at *p* < 0.05 for the Bonferroni adjustment and *p* < 0.001 for the kappa and weight kappa analyses.

## 3. Results

### 3.1. Feasibility

The final sample included was 212 children (10–12-years-old) and 62 adolescents (12–17-years -old). In general, participants understood the whole questionnaire without doubts. Children and adolescents spent an average of 15 ± 3.62 min and 9 ± 2.26 min respectively (including the distance and time question) to complete the whole questionnaire. A significant difference was found in the time to complete the questionnaire between the 10–11 age group (they lasted 15.13 ± 3.71 min) compared to the 12–13-year-old group, 14–15 years-old and 16–17-year-old groups (that lasted 10.65 ± 1.99 min, 9.78 ± 2.21 min, and 7.14 ± 2.08 min respectively, all *p* < 0.04). Differences in the time spent to complete the questionnaire in the tests by children and adolescents, age groups and sex are shown in Table 2. 

### 3.2. Reliability

The test-retest reliability analysis for children and adolescents is shown in Table 3. An extended version of Table 3, Table 4 and Table 5 can be seen in the supplemental Tables S1–S3. The Kappa values for children in “usual mode to school”, “usual mode from school”, “weekly mode to school”, “weekly mode from school”, “distance to school” and “time to school” reveals between light and almost perfect reliability (k = 0.19–0.88), while for adolescents it reveals between moderate and almost perfect reliability (k = 0.42–0.94). The metro/train response was excluded due to its non-existent use by the sample.

The reliability of “usual mode to school”, “usual mode from school”, “weekly mode to school”, “weekly mode from school”, “distance to school” and “time to school” by age groups is presented in Table 4. The reliability was between light and almost perfect for the groups between 6–7-years-old (k = 0.17–0.85), 8–9-years-old (k = 0.18–0.87), 10–11-years-old (k = 0.07–0.93), 12–13-years-old (k = 0.15–0.98) and 14–15-years-old (k = 0.07–0.91). The reliability was higher in the 16–17-year-olds (k = 0.56–1).

The reliability results of children and adolescents categorized by gender are shown in Table 5. In both children and adolescent groups, the kappa score was similar among boys and girls for the “usual mode to school”, “usual mode from school”, “distance to school” and “time to school”, that showed a moderate to almost perfect reliability (boy children; k = 0.63–0.93; girl children; k = 0.54–0.92; adolescents boys; k = 0.72–0.92 and adolescent girls; k = 0.82–0.96). In children, the reliability for “weekly mode to and from school” was lower in boys (k = 0.06–0.73) than in girls (k = 0.26–0.85). Similar results were shown in adolescent boys and girls for “weekly mode to and from school” items. Finally, the reliability for “weekly mode to school” and “weekly mode from school” was higher in adolescents boys (k = 0.49–0.81 and k = 0.49–0.82, respectively) than in boy children (k = 0.19−0.73 and k = 0.06−0.73, respectively).

## 4. Discussion

In this study, we examined the feasibility and reliability of the “New Version of Mode and Frequency of Commuting To and From School” questionnaire in a sample of Spanish children and adolescents. The reliability of the “New Version of Mode and Frequency of Commuting To and From School” questionnaire was shown as substantial for children and adolescents with very good feasibility in both, as well as by age groups and gender. Thus, our findings indicate that the “New Version of Mode and Frequency of Commuting To and From School” questionnaire is a feasible and reliable instrument to evaluate the ACS behaviour in Spanish children and adolescents.

The reason we evaluated feasibility was to analyze if: a) the questionnaire may fit during a normal lesson in the Spanish educational system which is 45 min in primary education and 60 min in secondary education, to make it feasible to schoolteachers from both primary and secondary educational levels, and secondly, b) to analyze the potential differences between children and adolescents and between age groups. The scientific literature shows that at certain ages, for instance children 10 years of age or younger, have difficulties completing a questionnaire appropriately, making this unreliable [30]. In addition, few studies have specifically analysed the feasibility of a questionnaire asking about the mode and frequency of ACS in young people. Most of the methodological studies about questionnaires focus more on validity and reliability issues, but they did not include feasibility, therefore, the present study tries to cover this gap regarding the scientific literature, through collecting the completion time and comprehension doubts about the questionnaire. In the current study, participants did not report any doubt to understand the questionnaire, and children and adolescents spent an average of 15 min and 9 min respectively to complete the whole questionnaire. The completion time of the questionnaire was somewhat long due to the questions required some reflection on their behaviors from the previous week. In a previous systematic review focused on self-report measurements to evaluate the mode and frequency of commuting to school, a total number of 158 studies were identified, where only two of them examined the feasibility [21]. In this first study, in 17 English adolescents from 13 to 15 years old, the time to complete travel to and from school was assessed with a questionnaire and a wearable camera [31]. The time required to complete the questionnaire and the doubts about it were not reported, hindering a possible comparison with our study. The second study was conducted in a Belgian sample of 146 children from 6 to 12 years old and forty-four parents; they completed a questionnaire to obtain a parental opinion on the perception of the intervention (i.e., to organise drop-off spots) and the time to complete it was not reported [32]. Once again, in both studies the results cannot be extrapolated to our study due to the disparity in the study sample and the measurement instruments. Future studies should investigate the feasibility of questionnaires assessing the mode, frequency, distance and time of commuting to and from school questionnaires in a larger and different sample of schools. In addition, an effort to avoid losing sample for future studies could be to complete the questionnaire online and the starting and ending time of it (as a feasibility data) would be remain automatically.

Regarding the reliability results, our study displayed correct and almost perfect reliability on average in the two previously validated questions (“usual mode to and from school”) and in the two additional questions (“distance and time to school”), while the reliability of the other two validated questions (“weekly mode to and from school”) was between light and almost perfect in children and adolescents, for age groups and gender. Similarly to our results, four studies showed reliability between 0.7 and 0.81 on average in all the questions focused on the mode of commuting to and from school [33,34,35,36]. In the first study, conducted in 152 Norwegian children from 12 to 13 years old, the Active Transportation to school and work in Norway questionnaire (ATN) obtained substantial reliability [33]. The second study carried out in 54 American children aged from 8 to 11 years old that completed the Survey on the Public Health Impacts of Children’s Travel to School questionnaire displayed substantial reliability [34]. In the third study, the Children’s Active Transportation and Independent Mobility questionnaire was used, reporting in 94 Canadian children substantial reliability [35]. Finally, the fourth study, the Take PART study (Physical Activity Research in Teenagers questionnaire) showed an almost perfect reliability in 626 German adolescents of 14 years old [36]. These results could indicate that the questionnaires evaluated previously, used concrete and direct questions to know the ACS behaviours and carried out a similar methodology (i.e., reliability with kappa and a 14-day test-retest protocol).

Concerning reliability in children and adolescents, our study showed substantial and almost perfect reliability on average in the “usual mode of commuting to and from school”, in “distance and in time to school”. In the “weekly mode to and from school” item our study showed light to almost perfect reliability in children and between correct and almost perfect reliability in adolescents. Four studies showed similar reliability results that our study (between substantial and almost perfect) in children and adolescents [33,37,38,39] and one study showed worse results compared to our study [35]. First, in a study conducted in German adolescents, the reliability of the mode to and from school was measured exhibiting a test-retest correlation of k = 0.93 [37]. A second study carried out in Ottawa children reported on the sum of trips to and from school a substantial to almost perfect reliability (ICC = 0.91) [38]. Third, the study of Carver et al. conducted in Melbourne adolescents showed a substantial reliability (ICC = 0.68) for the “weekly mode to school” item [39]. Fourthly, another study carried out in Norwegian children presented a moderate to good reliability for all usual modes of commuting, which was k > 0.81 for walking, cycling, car and public transport [33]. In relation to the item that assessed distance, a previous study obtained in Irish adolescents a substantial reliability (k = 0.7), finding slightly lower results compared to our study in the sample of adolescents (k = 0.9) [35]. The different reliability scores proved between the Irish study and our study could be due to the complexity and diversity of objectives of both questionnaires since in our case it only consists of six specific questions and in the Irish study, the questionnaire included several questions of various dimensions (aerobic fitness, PA, psychological and environmental determinants of PA) in adolescents. In summary, we only found lower reliability values in children compared to adolescents in the “weekly mode to and from school” item in our study. Due to the lower maturity development (i.e., cognitive) of children compared to adolescents, children may have less memory retention to report their mode of commuting [30]. In addition, the weekly patterns of the mode of commuting may change from one week (i.e., test) to the other (i.e., re-test), and it can be reflected in the lower reliability values. The good reliability found in the questionnaire showed that children and adolescents had a good comprehensibility of all the items. Furthermore, these provided us confidence about the suitability of the questionnaire to be used.

Regarding the reliability results by age groups, our study showed light reliability in the weekly mode to school in the 6–7-year-old, 8–9-year-old and 14–15-year-old groups. Same results offered in the “distance to school” and “time to school” items in the 6–7-year-old group. In the rest of age groups, the reliability was between correct and almost perfect in all items. Until the present date, there are no studies in the scientific literature that evaluate active commuting together in various age ranges. Thus, the user of the “New Version of Mode and Frequency of Commuting To and From School” questionnaire in children aged 6–7 years old is feasible and reliable, although it is important to take into account some tips: (1) more evaluators are needed than in older ages, (2) the time to complete the questionnaire is longer, (3) the reliability is lower compared to other older age groups.

Respect to the reliability results by gender in our study, we found lower results in boy children than in girl children in the “weekly mode to and from school” items. In the rest of the items (i.e., “usual mode to school”, “usual mode from school”, “distance to school” and “time to school”), the reliability was similar in boys and girls in children and adolescents. Several previous studies measured the reliability of the active commuting questionnaire by gender. For instance, in the study of Nelson et al., conducted in Irish adolescents (boys; *n* = 2083), a self-report questionnaire was used finding substantial reliability between items (k = 0.7 or above) and there were no gender differences in the reliability of the mode to and from school and distance compared to our study [35]. Similar results with respect to the gender were shown in a study conducted in British children where there were no gender differences in the reliability of the usual mode to and from school and distance [36]. In another study conducted in a sample of German adolescents (*n* = 626), the adolescents’ questionnaire about commuting mode was used displaying similar results regarding gender in the “time to school” and “usual mode of commuting to and from school” [37]. No differences in reliability between boys and girls in these studies could be because it is a completely assimilated and habitual behaviour in both genders.

This study has several limitations. First of all, only one city was studied, limiting the generalizability of the results to the whole population. Similar to the previous one, another limitation of this study was that schools were recruited by convenience. Another limitation is the loss of over 50% of the feasibility data in the sample, since the starting and/or ending time were missing and in addition, no other type of feasibility (cost, interpretability) measures were carried out. Another limitation may be a deliberate mistake or errors due to non-controllable external factors such as motivation, health state or a potential behaviour change between both measurements. Regarding strengths, most of the questions in the questionnaire have been previously validated [25] and the sample was expanded to very young children from 6–7 years old that provides novel information. Moreover, to our knowledge, this is the first study that evaluates the reliability of the questionnaire and it fulfills the important methodological criterion to evaluate with 8 - 14 days of difference between test and retest to minimize a learned response and observes reliability among age groups in an extensive range of ages [40]. In future studies, the “New Version of Mode and Frequency of Commuting To and From School” questionnaire should be reproduced in different places in Spain as well as in South American. In addition, it would be very important to know the reliability of the “New Version Mode and Frequency of Commuting To and From School” questionnaire in other languages to compare results between countries.

## 5. Conclusions

The “New Version of Mode and Frequency of Commuting To and From School” questionnaire is a feasible and reliable tool to evaluate the usual mode of commuting to and from school, the weekly mode of commuting to and from school, and the distance and time to school in Spanish children and adolescents. However, we must be cautious in relation to the feasibility results due to the diversity of limitations mentioned. The present study covers, with certain limitations, a gap in the scientific literature since there is a shortage of studies showing the reliability and especially the feasibility of the questionnaires on this topic.

## Figures and Tables

**Table 1:** Descriptive characteristics of the total sample and separately by children and adolescents.

	*n*	M ± SD/%
All		
Sex (male/female)	986	48.5/51.5
Age (year)	997	11.59 ± 2.98
Mother income (<1000 €/≥1000 €)	236/185	56.1/43.9
Father income (<1000 €/≥1000 €)	121/288	29.6/70.4
Children		
Sex (male/female)	311/324	48/52
Age (year)	635	9.77 ± 1.86
Mother income (<1000 €/≥1000 €)	218/171	56/44
Father income (<1000 €/≥ 1000 €)	108/267	28.8/71.2
Adolescents		
Sex (male/female)	176/184	49/51
Age (year)	362	14.80 ± 1.48
Mother income (<1000 €/≥1000 €)	18/14	56.3/43.8
Father income (<1000 €/≥1000 €)	13/21	38.2/61.8

Notes: *n*, sample size, M, mean, SD, standard deviation. The mother and father’s income were self-reported by them and corresponds to the monthly salary.

**Table 2:** Differences in the time spent in the tests by children and adolescents, age groups and gender.

	*n*	MD	SD	*p*
Children	101	15.02	3.62	0.00 *
Boys	48	16.23	4.41	0.22 *
Girls	53	14.15	2.85	0.13 *
Adolescents	184	8.89	2.26	0.00 *
Boys	90	9.56	2.12	0.64 *
Girls	94	8.23	1.96	0.73 *
Age Groups				
10–11	85	15.13	3.71	0.04 *
Boys	40	15.84	3.68	0.65 *
Girls	45	14.19	3.59	0.59 *
12–13	99	10.65	1.99	0.04 *
Boys	47	10.70	2.02	0.15 *
Girls	52	10.55	1.91	0.10 *
14–15	58	9.78	2.21	0.04 *
Boys	28	9.53	2.25	0.21 *
Girls	30	10.25	2.17	0.28 *
16–17	43	7.14	2.08	0.04 *
Boys	20	7.46	1.84	0.33 *
Girls	23	6.87	1.80	0.21

Notes: *n*, simple size. MD, mean deviation. SD, standard deviation. * *p* < 0.05.

**Table 3:** Test-retest reliability of the “New Version of Mode and Frequency of Commuting To and From School” questionnaire in children and adolescents.

	Children		Adolescents	
	*n*	Kappa	*n*	Kappa
Usual mode to school	495	0.88	303	0.91
Usual mode from school	498	0.83	303	0.94
Weekly mode to school				
Walk	635	0.60	362	0.63
Bike	635	0.20	362	0.62
Car	635	0.56	362	0.62
Motorbike	635	0.33	362	0.66
School bus	503	0.81	307	0.91
Public bus	635	0.45	362	0.69
Weekly mode from school				
Walk	635	0.56	362	0.67
Bike	635	0.19	362	0.51
Car	635	0.57	362	0.67
Motorbike	635	0.46	362	0.42
School bus	490	0.76	306	0.80
Public bus	635	0.53	362	0.76
Distance to school ^‡^	504	0.75	321	0.90
Time to school ^‡^	521	0.58	323	0.79
Total		0.48		0.60

Notes: *n*, sample size (children/adolescents). ^‡^, weighted kappa values. All *p* < 0.001. Extended version for Table 3 is available in supplemental Table S1.

**Table 4:** Test-retest reliability of the “New Version of Mode and Frequency of Commuting To and From School” questionnaire by age range.

	6–7 y *n* = 104	8–9 y *n* = 138	10–11 y *n* = 255	12–13 y *n* = 234	14–15 y *n* = 140	16–17 y *n* = 112
	Kappa	Kappa	Kappa	Kappa	Kappa	Kappa
Usual mode to school	0.85	0.87	0.93	0.98	0.87	0.84
Usual mode from school	0.84	0.81	0.91	0.94	0.91	0.93
Weekly mode to school						
Walk	0.50	0.58	0.61	0.67	0.57	0.63
Bike	0.31	0.18	0.29	0.25	0.58	0.71
Car	0.54	0.39	0.65	0.65	0.53	0.56
Motorbike	0.33	0.20	0.45	0.42	0.07	0.79
School bus	0.64	0.85	0.93	0.87	0.82	1
Public bus	0.17	0.26	0.38	0.79	0.67	0.67
Weekly mode from school						
Walk	0.44	0.44	0.60	0.71	0.63	0.60
Bike	0.39	0.20	0.07	0.15	0.52	0.71
Car	0.43	0.45	0.62	0.69	0.67	0.59
Motorbike	0.39	0.52	0.52	0.19	0.32	0.56
School bus	0.80	0.69	0.82	0.72	0.84	0.74
Public bus	0.22	0.40	0.59	0.76	0.79	0.70
Distance to school ^‡^	0.55	0.75	0.81	0.80	0.89	0.93
Time to school ^‡^	0.30	0.48	0.68	0.76	0.79	0.86
Total	0.38	0.40	0.54	0.61	0.55	0.59

Notes: y, years old. *n*, sample size. ^‡^, weighted kappa values. All *p* < 0.001. Extended version for Table 4 is available in supplemental Table S2.

**Table 5:** Test-retest reliability of the “New Version of Mode and Frequency of Commuting To and From School” questionnaire in children and adolescents questionnaire by gender.

	Children			Adolescents		
		Boys	Girls		Boys	Girls
	*n*	Kappa	Kappa	*n*	Kappa	Kappa
Usual mode to school	233/258	0.93	0.92	140/159	0.90	0.89
Usual mode from school	234/260	0.87	0.89	142/157	0.92	0.96
Weekly mode to school						
Walk	257/274	0.54	0.64	221/234	0.64	0.64
Bike	257/274	0.19	0.30	221/234	0.56	0.45
Car	257/274	0.53	0.59	221/234	0.63	0.59
Motorbike	257/274	0.19	0.52	221/234	0.49	0.62
School bus	203/217	0.73	0.85	189/199	0.81	0.92
Public bus	257/274	0.28	0.43	221/234	0.72	0.67
Weekly mode from school						
Walk	257/274	0.44	0.51	221/234	0.68	0.64
Bike	257/274	0.06	0.26	221/234	0.53	0.37
Car	257/274	0.57	0.53	221/234	0.73	0.61
Motorbike	257/274	0.43	0.50	221/234	0.49	0.35
School bus	194/213	0.73	0.79	187/200	0.82	0.73
Public bus	257/274	0.37	0.55	221/234	0.73	0.74
Distance to school ^‡^	244/259	0.78	0.71	155/164	0.90	0.89
Time to school ^‡^	249/271	0.63	0.54	155/166	0.76	0.82
Total		0.43	0.47		0.62	0.54

Notes: *n*, sample size (boys/girls). ^‡^, weighted kappa values. All *p* < 0.001. Extended version for Table 5 is available in supplemental Table S3.

## Supplementary Materials

The following are available online at https://www.mdpi.com/1660-4601/17/14/5039/s1, Figure S1. Sheet Register of the “New Version Mode and Frequency of commuting to and from school” questionnaire for Students’ doubts in Spanish version and Tables S1, S2 and S3. Test-retest reliability of the “New Version of Mode and Frequency of Commuting To and From School” questionnaire in children and adolescents, age groups and gender. Extended version.
