# Supplementary material for: Feasibility and Reliability of a Questionnaire to Assess the Mode, Frequency, Distance and Time of Commuting to and from School: The PACO Study
Source: Int J Environ Res Public Health. 2020 Jul 13;17(14):5039. doi: 10.3390/ijerph17145039 (PMC7399968; doi:10.3390/ijerph17145039)
Supplement: Supplementary file 1 [file ijerph-17-05039-s001.pdf]

Table S1. Test-retest reliability of the "New Version of Mode and Frequency of Commuting To and From School" questionnaire in children and adolescents. Extended version.

		Children *n* = 635		Adolescents *n* = 362
	n	Kappa	*n*	Kappa
Usual mode to school	495	0.88	303	0.91
Usual mode from school	498	0.83	303	0.94
Weekly mode to school				
Walk Monday	635	0.69	362	0.76
Bike Monday	635	0.11	362	0.69
Car Monday	635	0.66	362	0.75
Motorbike Monday	635	0.32	362	0.66
School bus Monday	525	0.91	318	0.96
Public bus Monday	635	0.57	362	0.80
Metro/train Monday	635	0.00	362	0.00
Others Monday	635	0.32	362	0.66
Walk Tuesday	635	0.66	362	0.66
Bike Tuesday	635	0.63	362	0.68
Car Tuesday	635	0.67	362	0.71
Motorbike Tuesday	635	0.45	362	1
School bus Tuesday	520	0.91	318	0.93
Public bus Tuesday	635	0.49	362	0.77
Metro/train Tuesday	635	−0.02	362	–
Others Tuesday	635	0.21	362	0.49
Walk Wednesday	635	0.66	362	0.71
Bike Wednesday	635	0.13	362	0.77
Car Wednesday	635	0.62	362	0.75
Motorbike Wednesday	635	0.49	362	1
School bus Wednesday	521	0.90	315	0.93
Public bus Wednesday	635	0.60	362	0.84
Metro/train Wednesday	635	–	362	–
Others Wednesday	635	0.56	362	0.49
Walk Thursday	635	0.68	362	0.74
Bike Thursday	635	0.12	362	0.77
Car Thursday	635	0.65	362	0.75
Motorbike Thursday	635	0.51	362	0.49
School bus Thursday	517	0.90	317	0.97
Public bus Thursday	635	0.55	362	0.79
Metro/train Thursday	635	0.00	362	–
Others Thursday	635	0.49	362	0.49
Walk Friday	635	0.66	362	0.72
Bike Friday	635	0.15	362	0.81
Car Friday	635	0.67	362	0.69
Motorbike Friday	635	0.46	362	0.49
School bus Friday	515	0.87	315	0.87
Public bus Friday	635	0.50	362	0.77
Metro/train Friday	635	−0.02	362	–
Others Friday	635	0.39	362	0.49
Weekly mode from school				

Walk Monday	635	0.67	362	0.73
Bike Monday	635	0.19	362	0.65
Car Monday	635	0.67	362	0.74
Motorbike Monday	635	0.60	362	0.85
School bus Monday	519	0.81	317	0.81
Public bus Monday	635	0.61	362	0.85
Metro/train Monday	635	0.00	362	–
Others Monday	635	0.39	362	0.32
Walk Tuesday	635	0.67	362	0.75
Bike Tuesday	635	−0.11	362	0.51
Car Tuesday	635	0.63	362	0.79
Motorbike Tuesday	635	0.66	362	0.56
School bus Tuesday	511	0.85	316	0.91
Public bus Tuesday	635	0.56	362	0.88
Metro/train Tuesday	635	−0.02	362	–
Others Tuesday	635	0.24	362	0.49
Walk Wednesday	635	0.65	362	0.77
Bike Wednesday	635	0.13	362	0.73
Car Wednesday	635	0.66	362	0.76
Motorbike Wednesday	635	0.58	362	0.24
School bus Wednesday	509	0.84	318	0.88
Public bus Wednesday	635	0.55	362	0.85
Metro/train Wednesday	635	–	362	–
Others Wednesday	635	0.28	362	0.49
Walk Thursday	635	0.69	362	0.72
Bike Thursday	635	0.13	362	0.65
Car Thursday	635	0.66	362	0.77
Motorbike Thursday	635	0.50	362	0.56
School bus Thursday	509	0.85	314	0.97
Public bus Thursday	635	0.48	362	0.81
Metro/train Thursday	635	−0.02	362	–
Others Thursday	635	0.21	362	0.66
Walk Friday	635	0.63	362	0.79
Bike Friday	635	0.18	362	0.62
Car Friday	635	0.64	362	0.70
Motorbike Friday	635	0.53	362	0.27
School bus Friday	500	0.81	311	0.88
Public bus Friday	635	0.54	362	0.81
Metro/train Friday	635	−0.02	362	–
Others Friday	635	0.24	362	0.49
Distance to school‡	504	0.75	321	0.90
Time to school‡	521	0.58	323	0.79

Notes: *n*, sample size (children/adolescents). ‡, weighted kappa values. All $p < 0.001$.

Table S2. Test-retest reliability of the "New Version of Mode and Frequency of Commuting To and From School questionnaire" by age range. Extended version.

	**6–7 Years old** *n* = 104	**8–9 Years old** *n* = 138	**10–11 Years old** *n* = 255	**12–13 Years old** *n* = 234	**14–15 Years old** *n* = 140	**16–17 Years old** *n* = 112
	**Kappa**	**Kappa**	**Kappa**	**Kappa**	**Kappa**	**Kappa**
Usual mode to school	0.85	0.87	0.93	0.98	0.87	0.84
Usual mode from school	0.84	0.81	0.91	0.94	0.91	0.93
Weekly mode to school						
Walk Monday	0.62	0.68	0.70	0.77	0.78	0.69
Bike Monday	0.23	−0.10	0.00	−0.06	0.65	0.85
Car Monday	0.55	0.49	0.76	0.80	0.74	0.62
Motorbike Monday	0.00	0.49	0.49	0.49	0.00	-
School bus Monday	0.74	0.84	1	0.96	1	1
Public bus Monday	0.16	0.31	0.51	0.92	0.86	0.76
Metro/train Monday	0.00	–	–	–	0.00	–
Others Monday	−0.01	0.00	0.66	1	0.39	0.00
Walk Tuesday	0.46	0.62	0.70	0.77	0.59	0.61
Bike Tuesday	0.00	−0.02	0.23	0.34	0.71	0.65
Car Tuesday	0.57	0.44	0.80	0.78	0.65	0.59
Motorbike Tuesday	−0.26	0.38	0.72	0.66	–	1
School bus Tuesday	0.74	0.92	0.96	0.96	0.92	1
Public bus Tuesday	0.16	0.13	0.45	0.86	0.79	0.72
Metro/train Tuesday	−0.13	–	–	–	–	–
Others Tuesday	−0.15	0.00	0.00	1	0.66	0.00
Walk Wednesday	0.63	0.62	0.65	0.76	0.65	0.65
Bike Wednesday	0.49	−0.10	−0.05	−0.09	0.79	0.85
Car Wednesday	0.62	0.42	0.69	0.78	0.71	0.63
Motorbike Wednesday	0.00	0.31	0.66	0.79	–	–
School bus Wednesday	0.74	0.84	0.96	0.96	0.92	1
Public bus Wednesday	0.19	0.43	0.57	0.89	0.88	0.80
Metro/train Wednesday	–	–	–	–	–	–
Others Wednesday	0.00	0.00	0.66	1	0.66	0.00
Walk Thursday	0.59	0.65	0.67	0.78	0.69	0.75
Bike Thursday	0.00	−0.01	0.39	0.38	1	0.74
Car Thursday	0.64	0.43	0.77	0.74	0.72	0.70
Motorbike Thursday	0.00	0.31	0.79	0.49	0.00	0.00
School bus Thursday	0.83	0.84	1	0.91	0.91	1
Public bus Thursday	0.19	0.20	0.50	0.88	0.78	0.75
Metro/train Thursday	0.00	–	–	–	–	–
Others Thursday	0.49	0.00	0.00	1	0.66	0.00
Walk Friday	0.62	0.65	0.65	0.74	0.68	0.70
Bike Friday	0.65	−0.02	−0.01	0.27	0.85	0.85
Car Friday	0.66	0.49	0.76	0.73	0.66	0.61
Motorbike Friday	0.00	0.31	0.65	0.49	–	0.00
School bus Friday	0.84	0.84	0.93	0.86	0.81	1
Public bus Friday	0.37	0.30	0.55	0.79	0.93	0.65
Metro/train Friday	−0.01	–	–	–	–	–
Others Friday	−0.01	0.00	0.66	1	0.66	0.00
Weekly mode from school						

Walk Monday	0.56	0.52	0.75	0.76	0.65	0.71
Bike Monday	0.38	0.00	−0.00	0.49	0.48	0.85
Car Monday	0.50	0.49	0.81	0.74	0.69	0.75
Motorbike Monday	0.31	0.79	0.72	0.00	0.79	1
School bus Monday	0.72	0.86	0.93	0.74	0.89	0.73
Public bus Monday	0.37	0.47	0.65	0.83	0.90	0.77
Metro/train Monday	0.00	–	–	–	–	–
Others Monday	−0.01	0.00	0.66	1	0.39	0.00
Walk Tuesday	0.67	0.48	0.70	0.81	0.68	0.70
Bike Tuesday	−0.01	−0.01	−0.01	−0.01	0.85	0.55
Car Tuesday	0.48	0.42	0.73	0.79	0.77	0.71
Motorbike Tuesday	0.38	0.74	0.74	–	0.00	1
School bus Tuesday	0.80	0.80	0.92	0.81	1	0.85
Public bus Tuesday	0.37	0.15	0.65	0.85	0.97	0.79
Metro/train Tuesday	−0.10	–	–	–	–	–
Others Tuesday	−0.10	0.00	−0.00	1	0.66	0.00
Walk Wednesday	0.65	0.47	0.68	0.77	0.72	0.73
Bike Wednesday	0.26	0.00	−0.05	0.32	0.74	0.85
Car Wednesday	0.56	0.47	0.74	0.81	0.72	0.69
Motorbike Wednesday	0.66	0.49	0.66	0.00	0.00	0.66
School bus Wednesday	0.80	0.80	0.89	0.81	1	0.73
Public bus Wednesday	0.42	0.28	0.59	0.79	0.92	0.80
Metro/train Wednesday	–	–	–	–	–	–
Others Wednesday	0.00	0.00	0.00	1	0.66	0.00
Walk Thursday	0.77	0.56	0.71	0.76	0.64	0.68
Bike Thursday	−0.01	0.49	−0.06	−0.01	0.85	0.85
Car Thursday	0.52	0.45	0.80	0.75	0.73	0.71
Motorbike Thursday	0.26	0.65	0.66	−0.04	0.66	0.49
School bus Thursday	0.80	0.86	0.89	0.85	1	1
Public bus Thursday	0.37	0.16	0.52	0.73	0.90	0.70
Metro/train Thursday	–	−0.01	–	–	–	–
Others Thursday	−0.01	0.00	−0.04	1	0.66	-
Walk Friday	0.68	0.46	0.66	0.74	0.67	0.75
Bike Friday	0.48	−0.15	−0.05	0.23	0.48	0.85
Car Friday	0.58	0.44	0.72	0.74	0.65	0.67
Motorbike Friday	0.49	0.74	0.49	−0.06	0.49	0.00
School bus Friday	0.80	0.78	0.85	0.82	0.94	0.73
Public bus Friday	0.37	0.25	0.55	0.80	0.91	0.71
Metro/train Friday	−0.01	–	–	–	–	–
Others Friday	−0.01	0.00	−0.04	1	0.66	0.00
Distance to school‡	0.55	0.75	0.81	0.80	0.89	0.93
Time to school‡	0.30	0.48	0.68	0.76	0.79	0.86

Notes: *n*, sample size. ‡, weighted kappa values. All $p < 0.001$.

Table S3. Test-retest reliability of the "New Version of Mode and Frequency of Commuting To and From School questionnaire" in children and adolescent by gender. Extended version.

	Children			Adolescents		
		Boys	Girls		Boys	Girls
	n	Kappa	Kappa	*n*	Kappa	Kappa
**Usual mode to school**	233/258	0.93	0.92	140/159	0.90	0.89
**Usual mode from school**	234/260	0.87	0.89	142/157	0.92	0.96
**Weekly mode to school**						
Walk Monday	302/324	0.66	0.73	176/184	0.80	0.73
Bike Monday	302/324	−0.17	0.32	176/184	0.65	0.79
Car Monday	302/324	0.65	0.68	176/184	0.74	0.76
Motorbike Monday	302/324	0.21	0.43	176/184	0.00	1
School bus Monday	251/273	0.88	0.92	153/164	1	0.93
Public bus Monday	302/324	0.54	0.60	176/184	0.79	0.80
Metro/train Monday	302/324	0.00	–	176/184	0.00	–
Others Monday	302/324	0.39	0.00	176/184	0.39	–
Walk Tuesday	302/324	0.63	0.69	176/184	0.69	0.64
Bike Tuesday	302/324	0.13	−0.01	176/184	0.81	0.32
Car Tuesday	302/324	0.67	0.67	176/184	0.70	0.71
Motorbike Tuesday	302/324	0.27	0.72	176/184	–	1
School bus Tuesday	249/270	0.87	0.92	153/164	0.93	0.93
Public bus Tuesday	302/324	0.54	0.42	176/184	0.78	0.74
Metro/train Tuesday	302/324	0.00	–	176/184	–	–
Others Tuesday	302/324	0.21	–	176/184	0.66	–
Walk Wednesday	302/324	0.63	0.69	176/184	0.71	0.72
Bike Wednesday	302/324	−0.11	0.27	176/184	0.79	0.74
Car Wednesday	302/324	0.60	0.65	176/184	0.74	0.75
Motorbike Wednesday	302/324	0.39	0.66	176/184	–	1
School bus Wednesday	249/271	0.95	0.87	176/184	0.93	0.93
Public bus Wednesday	302/324	0.69	0.53	176/184	0.81	0.87
Metro/train Wednesday	302/324	–	–	176/184	–	–
Others Wednesday	302/324	0.56	–	176/184	0.66	–
Walk Thursday	302/324	0.63	0.72	176/184	0.71	0.78
Bike Thursday	302/324	0.24	−0.11	176/184	0.81	0.66
Car Thursday	302/324	0.65	0.66	176/184	0.73	0.77
Motorbike Thursday	302/324	0.53	0.49	176/184	0.00	0.66
School bus Thursday	247/269	0.85	0.92	153/163	0.92	1
Public bus Thursday	302/324	0.64	0.44	176/184	0.81	0.75
Metro/train Thursday	302/324	–	–	176/184	–	–
Others Thursday	302/324	0.56	0.00	176/184	0.66	–
Walk Friday	302/324	0.58	0.75	176/184	0.72	0.71
Bike Friday	302/324	−0.13	0.24	176/184	0.82	0.79
Car Friday	302/324	0.00	0.63	176/184	0.65	0.72
Motorbike Friday	302/324	0.59	0.35	176/184	0.00	1
School bus Friday	246/268	0.81	0.90	153/160	0.86	0.88
Public bus Friday	302/324	0.62	0.47	176/184	0.73	0.82
Metro/train Friday	302/324	–	0.00	176/184	–	–
Others Friday	302/324	0.43	0.00	176/184	0.66	–
**Weekly mode from school**						
Walk Monday	302/324	0.68	0.67	176/184	0.77	0.70
Bike Monday	302/324	0.39	−0.07	176/184	0.70	0.49

Car Monday	302/324	0.68	0.66	176/184	0.77	0.72
Motorbike Monday	302/324	0.59	0.60	176/184	0.66	1
School bus Monday	245/273	0.77	0.83	152/164	0.88	0.76
Public bus Monday	302/324	0.52	0.66	176/184	0.86	0.83
Metro/train Monday	302/324	–	0.00	176/184	–	–
Others Monday	302/324	0.49	0.00	176/184	0.39	–
Walk Tuesday	302/324	0.65	0.69	176/184	0.77	0.73
Bike Tuesday	302/324	−0.13	−0.009	176/184	0.70	−0.01
Car Tuesday	302/324	0.64	0.61	176/184	0.75	0.83
Motorbike Tuesday	302/324	0.72	0.59	176/184	0.00	0.79
School bus Tuesday	244/266	0.80	0.87	152/163	0.93	0.89
Public bus Tuesday	302/324	0.52	0.58	176/184	0.86	0.90
Metro/train Tuesday	302/324	0.00	–	176/184	–	–
Others Tuesday	302/324	0.27	0.00	176/184	0.66	–
Walk Wednesday	302/324	0.64	0.66	176/184	0.78	0.77
Bike Wednesday	302/324	0.27	−0.009	176/184	0.82	0.56
Car Wednesday	302/324	0.67	0.65	176/184	0.76	0.76
Motorbike Wednesday	302/324	0.27	0.79	176/184	0.00	0.32
School bus Wednesday	241/267	0.83	0.83	152/165	0.93	0.84
Public bus Wednesday	302/324	0.45	0.61	176/184	0.84	0.85
Metro/train Wednesday	302/324	–	–	176/184	–	–
Others Wednesday	302/324	0.32	0.00	176/184	0.66	–
Walk Thursday	302/324	0.65	0.74	176/184	0.70	0.74
Bike Thursday	302/324	−0.11	0.27	176/184	0.70	0.49
Car Thursday	302/324	0.66	0.66	176/184	0.75	0.78
Motorbike Thursday	302/324	0.52	0.49	176/184	−0.08	1
School bus Thursday	240/268	0.83	0.85	149/164	0.00	0.70
Public bus Thursday	302/324	0.41	0.52	176/184	0.78	0.84
Metro/train Thursday	302/324	0.00	–	176/184	–	–
Others Thursday	302/324	0.27	0.00	176/184	1	–
Walk Friday	302/324	0.62	0.65	176/184	0.74	0.73
Bike Friday	302/324	0.16	0.21	176/184	0.76	0.32
Car Friday	302/324	0.66	0.63	176/184	0.76	0.64
Motorbike Wednesday	302/324	0.88	0.29	176/184	−0.00	0.49
School bus Friday	238/261	0.77	0.82	176/184	0.88	0.89
Public bus Friday	302/324	0.55	0.54	176/184	0.81	0.82
Metro/train Friday	302/324	–	0.00	176/184	–	–
Others Friday	302/324	0.32	0.00	176/184	0.66	–
**Distance to school‡**	244/259	0.78	0.71	155/164	0.90	0.89
**Time to school‡**	249/271	0.63	0.54	155/166	0.76	0.82

Notes: *n*, sample size (boys/girls). ‡, weighted kappa values. All $p < 0.001$.

Centro Educativo: ____ Curso: ____ Grupo: ___ Hoja de registro CUESTIONARIO *New Version of Mode and Frencuency of Commuting To and From School* Fecha de evaluación: ________________

ID	DATOS PERSONALES	P1	P2	P3	P4	P5	P6
TOTAL							

ANOTAR TIEMPO MEDIO DE REALIZACION:

Hora de inicio de entrada en clase:

Centro Educativo: ____ Curso: ____ Grupo: ___ Hoja de registro CUESTIONARIO *New Version of Mode and Frencuency of Commuting To and From School* Fecha de evaluación: ________________

Hora de inicio de realización de cuestionarios:

Hora de finalización de las 19 preguntas del cuestionario por cada alumno:

Hora de entrega del primer alumno que finaliza el cuestionario:

Hora de entrega del último alumno que finaliza el cuestionario:

ANOTAR LAS DUDAS DE LOS ESTUDIANTES:

SUGERENCIAS GENERALES PARA EVALUADORES:

- El evaluador deberá de ayudar a los estudiantes a completar el cuestionario correctamente (especialmente la primera página).
- Los estudiantes deben de rellenar las casillas así: ■ o donde aplique así: ● y en color negro o azul.
- El evaluador recordará a los alumnos que eviten hacer tachadura/señales/marcas en las casillas de respuesta.
- Si los estudiantes rellenan la casilla incorrectamente, ellos deben de hacer una flecha y escribir un no a la respuesta equivocada. Ej: NO
- El evaluador deberá de recordar a los estudiantes cuáles son las preguntas de múltiple respuesta.
- El evaluador deberá de revisar si los cuestionarios están correctamente rellenos cuando los entreguen.

Figure S1. Sheet Register of the "New Version of Mode and Frequency of commuting to and from school" questionnaire for Students' doubts in Spanish version.
